# Synthesis, crystal structure and thermal properties of poly[bis­[μ_2_-3-(amino­meth­yl)pyridine]­bis­(thio­cyanato)­cobalt(II)]

**DOI:** 10.1107/S2056989021003005

**Published:** 2021-03-26

**Authors:** Christoph Krebs, Inke Jess, Christian Näther

**Affiliations:** a Institut für Anorganische Chemie, Christian-Albrechts-Universität zu Kiel, Max-Eyth-Str. 2, D-24118 Kiel, Germany

**Keywords:** crystal structure, cobalt thio­cyanate, 3-(amino­meth­yl)pyridine, layer structure, thermal properties

## Abstract

The reaction of Co(NCS)_2_ with 3-(amino­meth­yl)pyridine as coligand leads to the formation of crystals of the title compound, [Co(NCS)_2_(C_6_H_8_N_2_)_2_]_*n*_, that were characterized by single-crystal X-ray analysis. In the crystal structure, the Co^II^ cations are octa­hedrally coordinated by two terminal N-bonded thio­cyanate anions as well as two pyridine and two amino N atoms of four symmetry-equivalent 3-(amino­meth­yl)pyridine coligands with all pairs of equivalent atoms in a *trans* position. The Co^II^ cations are linked by the 3-(amino­meth­yl)pyridine coligands into layers parallel to the *ac* plane. These layers are further linked by inter­molecular N—H⋯S hydrogen bonding into a three-dimensional network. The purity of the title compound was determined by X-ray powder diffraction and its thermal behavior was investigated by differential scanning calorimetry and thermogravimetry.

## Abstract 2

In the crystal structure of the title compound the Co^II^ cations are octa­hedrally coordinated by two terminal N-bonded thio­cyanate anions and four 3-(amino­meth­yl)pyridine coligands, of which two are coordinated through the pyridine N atom and two through the amino N atom. The cations are linked by the coligands into layers, that are further connected into a three-dimensional network by inter­molecular N—H⋯S hydrogen bonding.

## Chemical context   

Coordination compounds based on thio­cyanate anions show a variety of structures, that can be traced back to the versatile coordination behavior of this ligand (Buckingham, 1994 ▸, Wöhlert *et al.*, 2014 ▸; Werner *et al.*, 2015*a* ▸). Even if the majority of compounds contain only terminal N-bonded ligands, there is a large number of compounds in which the metal cations are linked by these anionic ligands into networks of different dimensionality (Đaković *et al.*, 2010 ▸; Kozísková *et al.*, 1990 ▸; Kabešová *et al.*, 1990 ▸; Prananto *et al.*, 2017 ▸; Suckert *et al.*, 2016 ▸; Wellm *et al.*, 2018 ▸). In those cases where the metal cations are octa­hedrally coordinated, different isomers can additionally be found, in which the metal cations are either all-*trans* or *cis*–*cis*–*trans* coordinated (Böhme *et al.*, 2020 ▸; Rams *et al.*, 2017 ▸). Which kind of compound is observed depends among other things on the nature of the metal cation, because the synthesis of compounds with bridging anionic ligands is easier with chalcophilic cations such as, for example, Cd^II^, whereas less chalcophilic metal cations such as Mn^II^, Fe^II^ and especially Co^II^ and Ni^II^ in several cases lead to the formation of compounds with terminal N-bonded thio­cyanate anions. This is of importance because this anionic ligand is able to mediate substantial magnetic exchange (Bassey *et al.*, 2020 ▸; Mekuimemba *et al.*, 2018 ▸; Palion-Gazda *et al.*, 2015 ▸; Mousavi *et al.*, 2020 ▸), which can lead to compounds that show a variety of magnetic properties. Co^II^ is of special importance because of its high magnetic anisotropy (Mautner *et al.*, 2018 ▸; Jochim *et al.*, 2020 ▸; Neumann *et al.*, 2019 ▸). This led to a renewed inter­est into compounds in which the metal cations are linked by these anionic ligands into chains or layers and an increasing number have been reported in the literature over the last decade (Jin *et al.*, 2007 ▸; Shi *et al.*, 2006 ▸; Mautner *et al.*, 2018 ▸).
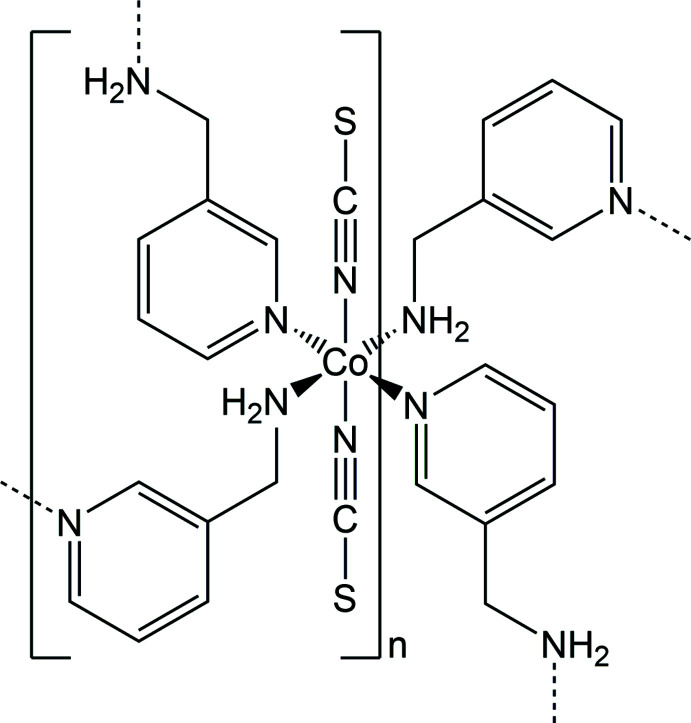


In our own investigations we are especially inter­ested in transition-metal thio­cyanate coordination polymers based on cobalt in which the metal cations are linked by μ-1,3-bridging anionic ligands into chains, because these compounds can show single-chain magnet (SCM) behavior. These are compounds in which the spins are ferromagnetically aligned along a chain with strong magnetic exchange within the chain and only weak inter­chain inter­actions to prevent 3D ordering (Sun *et al.*, 2010 ▸; Miyasaka *et al.*, 2005 ▸). In the course of this project we have prepared a large number of compounds with the general composition *M*(NCS)_2_(*L*)_2_ where *L* represents a pyridine derivative substituted at the 4-position (Werner *et al.*, 2015*b* ▸; Rams *et al.*, 2017 ▸, 2020 ▸). In principle, SCM behavior can also be observed in 2D compounds if the ferromagnetic chains are linked into layers by bridging ligands that do not mediate strong magnetic exchange. Therefore, we became inter­ested in 3-(amino­meth­yl)pyridine as it can coordinate to metal cations *via* the pyridine and the amino N atom and for which no cobalt(II) thio­cyanate compounds had been reported. Therefore, we reacted Co(NCS)_2_ with 3-(amino­meth­yl)pyridine in different molar ratios, which always led to the formation of crystalline powders with the composition Co(NCS)_2_(3-(amino­methyl­pyridine)_2_ (see *Synthesis and crystallization*). This composition indicated that either the organic coligand does not bridge neighboring metal centers or that only terminal-coordinated thio­cyanate anions are present. IR spectroscopic measurements reveal that the CN stretching vibration of the anionic ligand is observed at 2077cm^−1^, which points to the presence of terminal N-bonded anionic ligands (Fig. S1). To prove these assumptions, single crystals were grown and characterized by single-crystal X-ray diffraction, which proves that this crystalline phase is isotypic to the corresponding Cd compound already reported in the literature, in which the Cd^II^ or Co^II^ cations are linked into layers by the 3-(amino­meth­yl)pyridine ligands (see *Structural commentary*). Comparison of the experimental X-ray powder pattern with that calculated from the single-crystal data proves that a pure crystalline phase has been obtained (Fig. S2). For the more chalcophilic Cd^II^ cations another compound with the composition Cd(NCS)_2_(3-(amino­methyl­pyridine) is known, in which the Cd^II^ cations are linked by bridging anionic ligands. With Co(NCS)_2_ we found no access to this compound in solution and, therefore, we tried to prepare a 3-(amino­meth­yl)pyridine-deficient phase by thermal ligand removal from the title compound. Therefore, the title compound was investigated by thermogravimetry coupled to differential scanning calorimetry (TG-DSC). Upon heating at a rate of 8°C min^−1^ the compound starts to decompose at about 215°C and upon further heating a steady mass loss with no discrete decomposition events is observed (Fig. S3). To increase the resolution a second TG-DSC measurement with 1°C min^−1^ was performed, which does not improve the resolution significantly (Fig. S4). Based on these measurements, there is no indication for the formation of another currently unknown 3-(amino­meth­yl)pyridine-deficient compound.

## Structural commentary   

**Figure 1:**
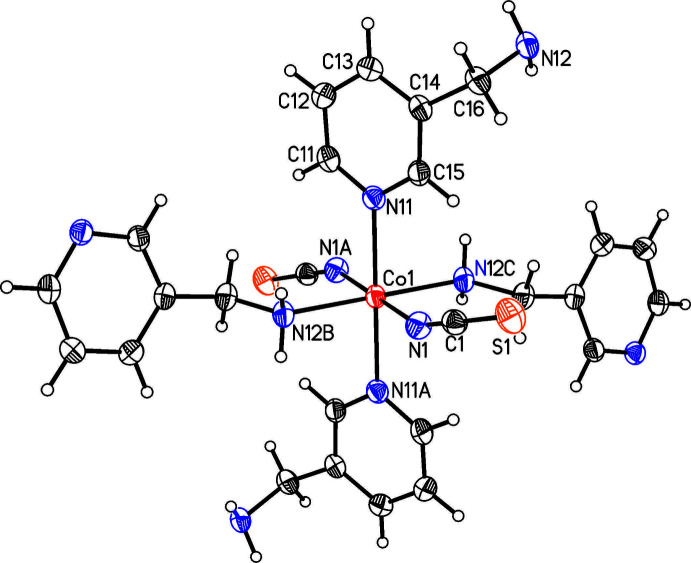
Crystal structure of the title compound with labeling and displacement ellipsoids drawn at the 50% probability level. Symmetry codes: A = −*x*, −*y* + 1, −*z*, B = 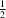 − *x*, −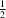 − *y*, 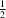 − *z*, C = −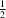 + *x*, 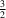 − *y*, −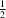 + *z*. Color code: Co (red), N (blue) and S (orange).

**Figure 2:**
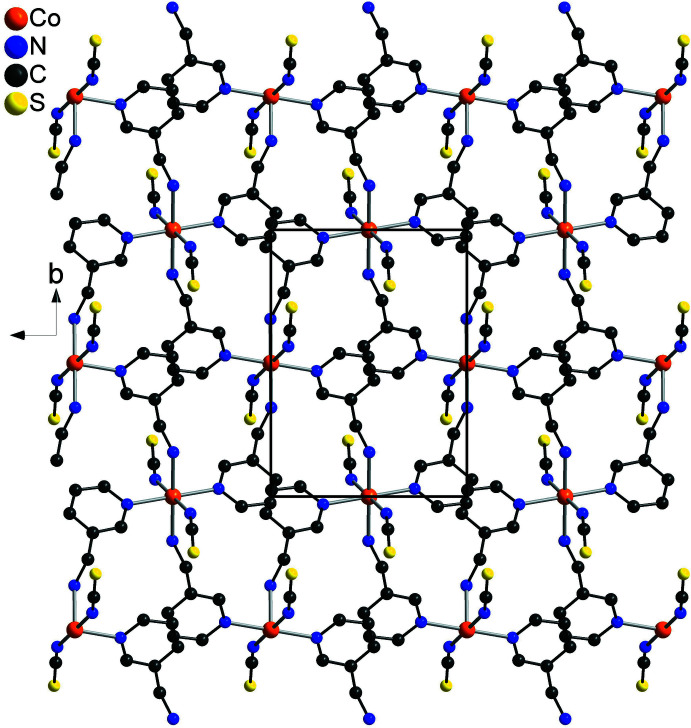
Crystal structure of the title compound viewed along the crystallographic *a* axis. The H atoms are omitted for clarity.

The asymmetric unit of the title compound, Co(NCS)_2_(C_6_H_8_N_2_)_2_, consists of one Co^II^ cation that is located on a center of inversion as well as one thio­cyanate anion and one 3-(amino­meth­yl)pyridine coligand in general positions (Fig. 1 ▸). The Co^II^ cations are sixfold coordinated by two symmetry-equivalent terminal N-bonded anionic ligands as well as four symmetry-equivalent 3-(amino­meth­yl)pyridine coligands, of which two are coordinated through the pyridine N atom and two through the amino N atom to the cations, with each pair of identical atoms in the *trans* position to each other (Fig. 1 ▸). The Co—N bond lengths to the amino N atom are significantly shorter than those to the pyridine N atoms, indicating that this is the stronger inter­action (Table 1 ▸). The bond angles around the Co^II^ centers deviate by less than 1.95 (6)° from the ideal values, which indicates that the octa­hedra are only slightly distorted (Table 1 ▸). This is also obvious from the octa­hedral angle variance of 1.6 and the mean octa­hedral quadratic elongation of 1.001 calculated using the method of Robinson (Robinson *et al.*, 1971 ▸). The Co cations are linked by bridging 3-(amino­meth­yl)pyridine ligands into layers that are parallel to the *bc* plane (Fig. 2 ▸). These layers are constructed of large rings that consist of four Co^II^ cations and four 3-(amino­meth­yl)pyridine coligands (Fig. 2 ▸).

## Supra­molecular features   

**Figure 3:**
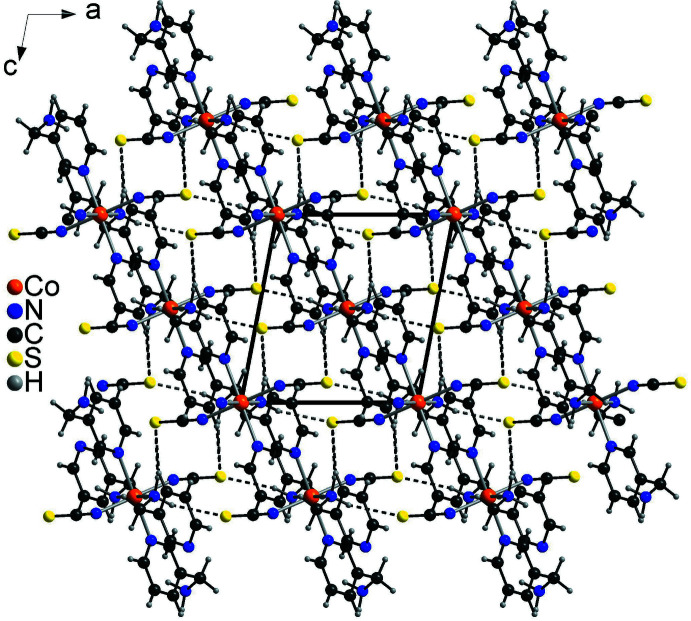
Crystal structure of the title compound viewed along the crystallographic *b* axis. Inter­molecular N—H⋯S hydrogen bonds are shown as dashed lines.

The Co(NCS)_2_ layers are arranged in stacks that elongate along the crystallographic *a*-axis direction (Fig. 2 ▸). The layers are linked into a three-dimensional network by inter­molecular N—H⋯S hydrogen bonding between the thio­cyanate S atoms and the amino H atoms, in which the S atoms act as acceptors for two of these hydrogen bonds (Fig. 3 ▸ and Table 2 ▸). The N—H⋯S angles are close to linear, which indicates that this is a strong inter­action. There are additional C—H⋯S and C—H⋯N intra- and inter­molecular inter­actions, but their geometrical parameters indicate that these are not strong inter­actions (Table 2 ▸).

## Database survey   

In the Cambridge Structural Database (CSD version 5.42, last update November 2020; Groom *et al.*, 2016 ▸) no cobalt thio­cyanate compounds with 3-(amino­meth­yl)pyridine as coligand are reported. However, some compounds based on Zn(NCS)_2_ and Cd(NCS)_2_ are published, in which the cations are always octa­hedrally coordinated (Neumann *et al.*, 2017 ▸). This includes Cd(NCS)_2_[3-(amino­meth­yl)pyridine]_2_-tris­[3-(amino­meth­yl)]pyridine solvate (QEKYOX), in which the Cd^II^ cations are also linked into layers, that contain large pores, in which additional 3-(amino­meth­yl)pyridine solvate mol­ecules are embedded. The same report also describes *M*(NCS)_2_[3-(amino­meth­yl)pyridine]_2_ [*M* = Cd (QEKZEO), Zn (QEKYUD)], which is isotypic to the title compound. Finally, two compounds with the composition *M*(NCS)_2_[3-(amino­meth­yl)pyridine] [*M* = Cd (QEKZIS), Zn (QEKZAK)] are reported. The Zn compound consists of dimers, in which each two Zn^II^ cations are linked by each two 3-(amino­meth­yl)pyridine ligands. In contrast, in the crystal structure of the Cd compound, the Cd^II^ cations are linked into chains by the 3-(amino­meth­yl)pyridine ligands that are further connected into layers by μ-1,3-bridging thio­cyanate anions. This compound is the only one which shows an *cis*–*cis*–*trans* coordination of the metal cations.

## Synthesis and crystallization   

**Experimental details**

Elemental analysis was performed using a EURO EA elemental analyzer fabricated by EURO VECTOR Instruments. The IR spectrum was measured using an ATI Mattson Genesis Series FTIR Spectrometer, control software: WINFIRST, from ATI Mattson. The PXRD measurement was performed with Cu *K*α_1_ radiation (λ = 1.540598 Å) using a Stoe Transmission Powder Diffraction System (STADI P) that is equipped with a MYTHEN 1K detector and a Johansson-type Ge(111) monochromator. Thermogravimetry and differential scanning calorimetry (TG-DSC) measurements were performed in a dynamic nitro­gen atmosphere in Al_2_O_3_ crucibles using a STA-PT 1600 thermobalance from Linseis. The instrument was calibrated using standard reference materials.

**Synthesis**

3-(Amino­meth­yl)pyridine and Co(NCS)_2_ were purchased from Alfa Aesar. All chemicals were used without further purification. Single crystals were obtained by reacting 1 mmol Co(NCS)_2_ (175.1 mg) with 0.2 mmol 3-(amino­meth­yl)pyridine (216.3 mg) in 3 mL of ethanol. After approximately one week blue-colored crystals were obtained, which were suitable for single crystal X-ray analysis. For the synthesis of crystalline powders the same amounts of reactants were stirred in 1 mL of ethanol for 3 d. The blue-colored precipitate was filtered and dried in air. Yield: 70%. Elemental analysis calculated for C_14_H_16_N_6_CoS_2_ (391.4 g mol^−1^) C 42.96%, H 4.12%, N 21.47%, S 16.39%, found: C 42.82%, H 4.01%, N 21.32%, S 16.29%. IR: ν = 3282 (*m*), 3245 (*m*), 3161 (*w*), 3058 (*w*), 3049 (*w*), 2979 (*w*), 2955 (*sh*), 2946 (*w*), 2874 (*vw*), 2862 (*sh*), 2077 (*vs*), 2033 (*w*), 1603 (*sh*), 1595 (*m*), 1582 (*m*), 1480 (*m*), 1447 (*w*), 1429 (*m*), 1361 (*w*), 1344 (*w*), 1333 (*w*), 1248 (*w*), 1229 (*w*), 1191 (*m*), 1150 (*m*), 1136 (*s*), 1103 (*m*), 1053 (*m*), 1039 (*w*), 990 (*s*), 965 (*m*), 943 (*w*), 933 (*m*), 895 (*w*), 852 (*m*), 841 (*sh*), 807 (*s*), 785 (*m*), 715 (*s*), 646 (*m*), 628 (*m*), 568 (*s*), 509 (*w*) cm^−1^.

## Refinement   

Crystal data, data collection and structure refinement details are summarized in Table 3 ▸. All non-hydrogen atoms were refined anisotropically. C—H and N—H H atoms were located in difference maps but positioned with idealized geometry and refined isotropically with *U*_iso_(H) = 1.2*U*_eq_(C) (1.5 for amino H atoms) using a riding model.

## Supplementary Material

Crystal structure: contains datablock(s) I. DOI: 10.1107/S2056989021003005/zl5009sup1.cif

Structure factors: contains datablock(s) I. DOI: 10.1107/S2056989021003005/zl5009Isup2.hkl

IR spectra of the title compound. The value of the CN stretching vibration is given. DOI: 10.1107/S2056989021003005/zl5009sup3.png

Experimental (A) and calculated X-ray powder pattern (B) of the title compound. For the calculation of the powder pattern the lattice parameters obtained from a Pawley fit of a powder pattern measured at room temperature were used. DOI: 10.1107/S2056989021003005/zl5009sup4.png

DTG, TG and DSC curve of the title compound measured with 8C/min. DOI: 10.1107/S2056989021003005/zl5009sup5.png

DTG, TG and DSC curve of the title compound measured with 1C/min. DOI: 10.1107/S2056989021003005/zl5009sup6.png

CCDC reference: 2072509

Additional supporting information:  crystallographic information; 3D view; checkCIF report

## Figures and Tables

**Table 1:** Selected geometric parameters (Å, °)

Co1—N1	2.1038 (16)	Co1—N11	2.2107 (15)
Co1—N2^i^	2.1821 (15)		
			
N1^ii^—Co1—N1	180.00 (8)	N1—Co1—N11	89.41 (6)
N1—Co1—N2^iii^	91.95 (6)	N2^iii^—Co1—N11	89.67 (6)
N1—Co1—N2^i^	88.05 (6)	N2^i^—Co1—N11	90.33 (6)
N1^ii^—Co1—N11	90.59 (6)	N11—Co1—N11^ii^	180.0

Symmetry codes: (i) -x+{\script{1\over 2}}, y-{\script{1\over 2}}, -z+{\script{1\over 2}}; (ii) -x, -y+1, -z; (iii) x-{\script{1\over 2}}, -y+{\script{3\over 2}}, z-{\script{1\over 2}}.

**Table 2:** Hydrogen-bond geometry (Å, °)

*D*—H⋯*A*	*D*—H	H⋯*A*	*D*⋯*A*	*D*—H⋯*A*
C11—H11⋯N1^ii^	0.95	2.69	3.207 (3)	115
C12—H12⋯S1^i^	0.95	2.93	3.696 (2)	138
C15—H15⋯N1	0.95	2.66	3.163 (2)	114
N2—H1*N*2⋯S1^iv^	0.91	2.87	3.7430 (17)	162
N2—H2*N*2⋯S1^v^	0.91	2.65	3.5044 (17)	157

Symmetry codes: (i) -x+{\script{1\over 2}}, y-{\script{1\over 2}}, -z+{\script{1\over 2}}; (ii) -x, -y+1, -z; (iv) x, y, z+1; (v) x-{\script{1\over 2}}, -y+{\script{3\over 2}}, z+{\script{1\over 2}}.

**Table 3:** Experimental details

Crystal data	
Chemical formula	[Co(NCS)_2_(C_6_H_8_N_2_)_2_]
*M*_r_	391.38
Crystal system, space group	Monoclinic, *P*2_1_/*n*
Temperature (K)	200
*a*, *b*, *c* (Å)	8.2442 (4), 11.9186 (4), 8.9204 (4)
β (°)	100.807 (4)
*V* (Å^3^)	860.97 (6)
*Z*	2
Radiation type	Mo *K*α
μ (mm^−1^)	1.25
Crystal size (mm)	0.20 × 0.15 × 0.12

Data collection	
Diffractometer	STOE IPDS2
Absorption correction	Numerical (*X-AREA*; Stoe & Cie, 2002 ▸)
*T*_min_, *T*_max_	0.709, 0.886
No. of measured, independent and observed [*I* > 2σ(*I*)] reflections	13295, 1871, 1702
*R*_int_	0.029
(sin θ/λ)_max_ (Å^−1^)	0.638

Refinement	
*R*[*F* ^2^ > 2σ(*F* ^2^)], *wR*(*F* ^2^), *S*	0.029, 0.071, 1.15
No. of reflections	1871
No. of parameters	106
H-atom treatment	H-atom parameters constrained
Δρ_max_, Δρ_min_ (e Å^−3^)	0.30, −0.23

Computer programs: *X-AREA* (Stoe & Cie, 2002 ▸), *SHELXS97* (Sheldrick, 2008 ▸), *SHELXL2016/6* (Sheldrick, 2015 ▸), *DIAMOND* (Brandenburg & Putz, 1999 ▸) and *publCIF* (Westrip, 2010 ▸).

## supplementary crystallographic information

### Crystal data

**Table:**

[Co(NCS)_2_(C_6_H_8_N_2_)_2_]	*F*(000) = 402
*M**_r_* = 391.38	*D*_x_ = 1.510 Mg m^−^^3^
Monoclinic, *P*2_1_/*n*	Mo *K*α radiation, λ = 0.71073 Å
*a* = 8.2442 (4) Å	Cell parameters from 13295 reflections
*b* = 11.9186 (4) Å	θ = 2.9–27.0°
*c* = 8.9204 (4) Å	µ = 1.25 mm^−^^1^
β = 100.807 (4)°	*T* = 200 K
*V* = 860.97 (6) Å^3^	Block, light blue
*Z* = 2	0.20 × 0.15 × 0.12 mm

### Data collection

**Table:**

STOE IPDS-2 diffractometer	1702 reflections with *I* > 2σ(*I*)
ω scans	*R*_int_ = 0.029
Absorption correction: numerical (X-AREA; Stoe & Cie, 2002)	θ_max_ = 27.0°, θ_min_ = 2.9°
*T*_min_ = 0.709, *T*_max_ = 0.886	*h* = −10→10
13295 measured reflections	*k* = −15→15
1871 independent reflections	*l* = −11→11

### Refinement

**Table:**

Refinement on *F*^2^	Primary atom site location: structure-invariant direct methods
Least-squares matrix: full	Secondary atom site location: difference Fourier map
*R*[*F*^2^ > 2σ(*F*^2^)] = 0.029	Hydrogen site location: inferred from neighbouring sites
*wR*(*F*^2^) = 0.071	H-atom parameters constrained
*S* = 1.15	*w* = 1/[σ^2^(*F*_o_^2^) + (0.0359*P*)^2^ + 0.3351*P*] where *P* = (*F*_o_^2^ + 2*F*_c_^2^)/3
1871 reflections	(Δ/σ)_max_ < 0.001
106 parameters	Δρ_max_ = 0.30 e Å^−^^3^
0 restraints	Δρ_min_ = −0.22 e Å^−^^3^

### Special details

**Table:**

Geometry. All esds (except the esd in the dihedral angle between two l.s. planes) are estimated using the full covariance matrix. The cell esds are taken into account individually in the estimation of esds in distances, angles and torsion angles; correlations between esds in cell parameters are only used when they are defined by crystal symmetry. An approximate (isotropic) treatment of cell esds is used for estimating esds involving l.s. planes.

### Fractional atomic coordinates and isotropic or equivalent isotropic displacement parameters (Å^2^)

**Table:**

	*x*	*y*	*z*	*U*_iso_*/*U*_eq_	
Co1	0.000000	0.500000	0.000000	0.02255 (11)	
N1	0.2001 (2)	0.56371 (13)	−0.08934 (18)	0.0295 (3)	
C1	0.3105 (2)	0.62263 (15)	−0.0971 (2)	0.0267 (4)	
S1	0.46298 (6)	0.70951 (4)	−0.10573 (7)	0.03779 (14)	
N11	0.14044 (19)	0.52941 (12)	0.23318 (17)	0.0259 (3)	
C11	0.1228 (2)	0.46286 (15)	0.3504 (2)	0.0293 (4)	
H11	0.044225	0.403768	0.332043	0.035*	
C12	0.2131 (3)	0.47585 (16)	0.4966 (2)	0.0314 (4)	
H12	0.198637	0.425522	0.575614	0.038*	
C13	0.3246 (2)	0.56334 (16)	0.5256 (2)	0.0304 (4)	
H13	0.388294	0.573805	0.625018	0.036*	
C14	0.3425 (2)	0.63566 (14)	0.4080 (2)	0.0260 (4)	
C15	0.2496 (2)	0.61412 (14)	0.2644 (2)	0.0266 (4)	
H15	0.263911	0.662217	0.183003	0.032*	
C16	0.4576 (2)	0.73543 (15)	0.4332 (2)	0.0296 (4)	
H16A	0.561774	0.712435	0.500465	0.035*	
H16B	0.484490	0.758046	0.333897	0.035*	
N2	0.38938 (19)	0.83381 (12)	0.50202 (18)	0.0271 (3)	
H1N2	0.389470	0.816364	0.601345	0.032*	
H2N2	0.281758	0.840369	0.455359	0.032*	

### Atomic displacement parameters (Å^2^)

**Table:**

	*U*^11^	*U*^22^	*U*^33^	*U*^12^	*U*^13^	*U*^23^
Co1	0.02203 (18)	0.01854 (17)	0.02615 (17)	−0.00132 (12)	0.00208 (12)	0.00035 (12)
N1	0.0261 (8)	0.0300 (8)	0.0322 (8)	−0.0025 (6)	0.0051 (6)	0.0014 (6)
C1	0.0269 (9)	0.0261 (8)	0.0271 (9)	0.0034 (7)	0.0047 (7)	−0.0018 (7)
S1	0.0301 (3)	0.0337 (3)	0.0503 (3)	−0.0084 (2)	0.0094 (2)	−0.0046 (2)
N11	0.0263 (8)	0.0229 (7)	0.0275 (7)	−0.0014 (6)	0.0021 (6)	−0.0015 (6)
C11	0.0305 (10)	0.0230 (8)	0.0345 (10)	−0.0039 (7)	0.0063 (8)	−0.0023 (7)
C12	0.0375 (11)	0.0277 (9)	0.0289 (9)	−0.0021 (7)	0.0065 (8)	0.0028 (7)
C13	0.0348 (10)	0.0289 (9)	0.0263 (9)	−0.0005 (8)	0.0031 (7)	−0.0027 (7)
C14	0.0260 (9)	0.0212 (8)	0.0307 (9)	0.0004 (7)	0.0052 (7)	−0.0038 (7)
C15	0.0289 (9)	0.0210 (8)	0.0289 (9)	−0.0008 (7)	0.0033 (7)	0.0004 (6)
C16	0.0282 (9)	0.0248 (9)	0.0350 (9)	−0.0028 (7)	0.0042 (8)	−0.0045 (7)
N2	0.0270 (8)	0.0208 (7)	0.0326 (8)	−0.0016 (6)	0.0036 (6)	−0.0010 (6)

### Geometric parameters (Å, º)

**Table:**

Co1—N1^i^	2.1038 (16)	C12—C13	1.382 (3)
Co1—N1	2.1038 (16)	C12—H12	0.9500
Co1—N2^ii^	2.1821 (15)	C13—C14	1.387 (3)
Co1—N2^iii^	2.1821 (15)	C13—H13	0.9500
Co1—N11	2.2107 (15)	C14—C15	1.388 (3)
Co1—N11^i^	2.2107 (15)	C14—C16	1.511 (2)
N1—C1	1.162 (2)	C15—H15	0.9500
C1—S1	1.6415 (19)	C16—N2	1.482 (2)
N11—C11	1.342 (2)	C16—H16A	0.9900
N11—C15	1.346 (2)	C16—H16B	0.9900
C11—C12	1.384 (3)	N2—H1N2	0.9100
C11—H11	0.9500	N2—H2N2	0.9100
			
N1^i^—Co1—N1	180.00 (8)	C13—C12—H12	120.6
N1^i^—Co1—N2^ii^	88.05 (6)	C11—C12—H12	120.6
N1—Co1—N2^ii^	91.95 (6)	C12—C13—C14	119.24 (18)
N1^i^—Co1—N2^iii^	91.95 (6)	C12—C13—H13	120.4
N1—Co1—N2^iii^	88.05 (6)	C14—C13—H13	120.4
N2^ii^—Co1—N2^iii^	180.0	C13—C14—C15	117.69 (17)
N1^i^—Co1—N11	90.59 (6)	C13—C14—C16	121.98 (17)
N1—Co1—N11	89.41 (6)	C15—C14—C16	120.33 (16)
N2^ii^—Co1—N11	89.67 (6)	N11—C15—C14	124.18 (17)
N2^iii^—Co1—N11	90.33 (6)	N11—C15—H15	117.9
N1^i^—Co1—N11^i^	89.41 (6)	C14—C15—H15	117.9
N1—Co1—N11^i^	90.59 (6)	N2—C16—C14	114.06 (15)
N2^ii^—Co1—N11^i^	90.33 (6)	N2—C16—H16A	108.7
N2^iii^—Co1—N11^i^	89.67 (6)	C14—C16—H16A	108.7
N11—Co1—N11^i^	180.0	N2—C16—H16B	108.7
C1—N1—Co1	156.84 (15)	C14—C16—H16B	108.7
N1—C1—S1	177.97 (17)	H16A—C16—H16B	107.6
C11—N11—C15	116.60 (16)	C16—N2—Co1^iv^	121.54 (11)
C11—N11—Co1	121.68 (12)	C16—N2—H1N2	106.9
C15—N11—Co1	121.71 (12)	Co1^iv^—N2—H1N2	106.9
N11—C11—C12	123.39 (17)	C16—N2—H2N2	106.9
N11—C11—H11	118.3	Co1^iv^—N2—H2N2	106.9
C12—C11—H11	118.3	H1N2—N2—H2N2	106.7
C13—C12—C11	118.85 (17)		
			
C15—N11—C11—C12	−1.8 (3)	Co1—N11—C15—C14	−179.17 (13)
Co1—N11—C11—C12	177.39 (15)	C13—C14—C15—N11	1.8 (3)
N11—C11—C12—C13	1.7 (3)	C16—C14—C15—N11	−177.78 (17)
C11—C12—C13—C14	0.2 (3)	C13—C14—C16—N2	−79.6 (2)
C12—C13—C14—C15	−1.8 (3)	C15—C14—C16—N2	100.0 (2)
C12—C13—C14—C16	177.72 (17)	C14—C16—N2—Co1^iv^	−164.90 (12)
C11—N11—C15—C14	0.0 (3)		

Symmetry codes: (i) −*x*, −*y*+1, −*z*; (ii) *x*−1/2, −*y*+3/2, *z*−1/2; (iii) −*x*+1/2, *y*−1/2, −*z*+1/2; (iv) −*x*+1/2, *y*+1/2, −*z*+1/2.

### Hydrogen-bond geometry (Å, º)

**Table:**

*D*—H···*A*	*D*—H	H···*A*	*D*···*A*	*D*—H···*A*
C11—H11···N1^i^	0.95	2.69	3.207 (3)	115
C12—H12···S1^iii^	0.95	2.93	3.696 (2)	138
C15—H15···N1	0.95	2.66	3.163 (2)	114
N2—H1*N*2···S1^v^	0.91	2.87	3.7430 (17)	162
N2—H2*N*2···S1^vi^	0.91	2.65	3.5044 (17)	157

Symmetry codes: (i) −*x*, −*y*+1, −*z*; (iii) −*x*+1/2, *y*−1/2, −*z*+1/2; (v) *x*, *y*, *z*+1; (vi) *x*−1/2, −*y*+3/2, *z*+1/2.
