# Supplementary figures and images for: Synthesis, crystal structure and thermal properties of poly[bis­[μ2-3-(amino­meth­yl)pyridine]­bis­(thio­cyanato)­cobalt(II)]
Source: Acta Crystallogr E Crystallogr Commun. 2021 Mar 26;77(Pt 4):428–32. doi: 10.1107/S2056989021003005 (PMC8025872; doi:10.1107/S2056989021003005)

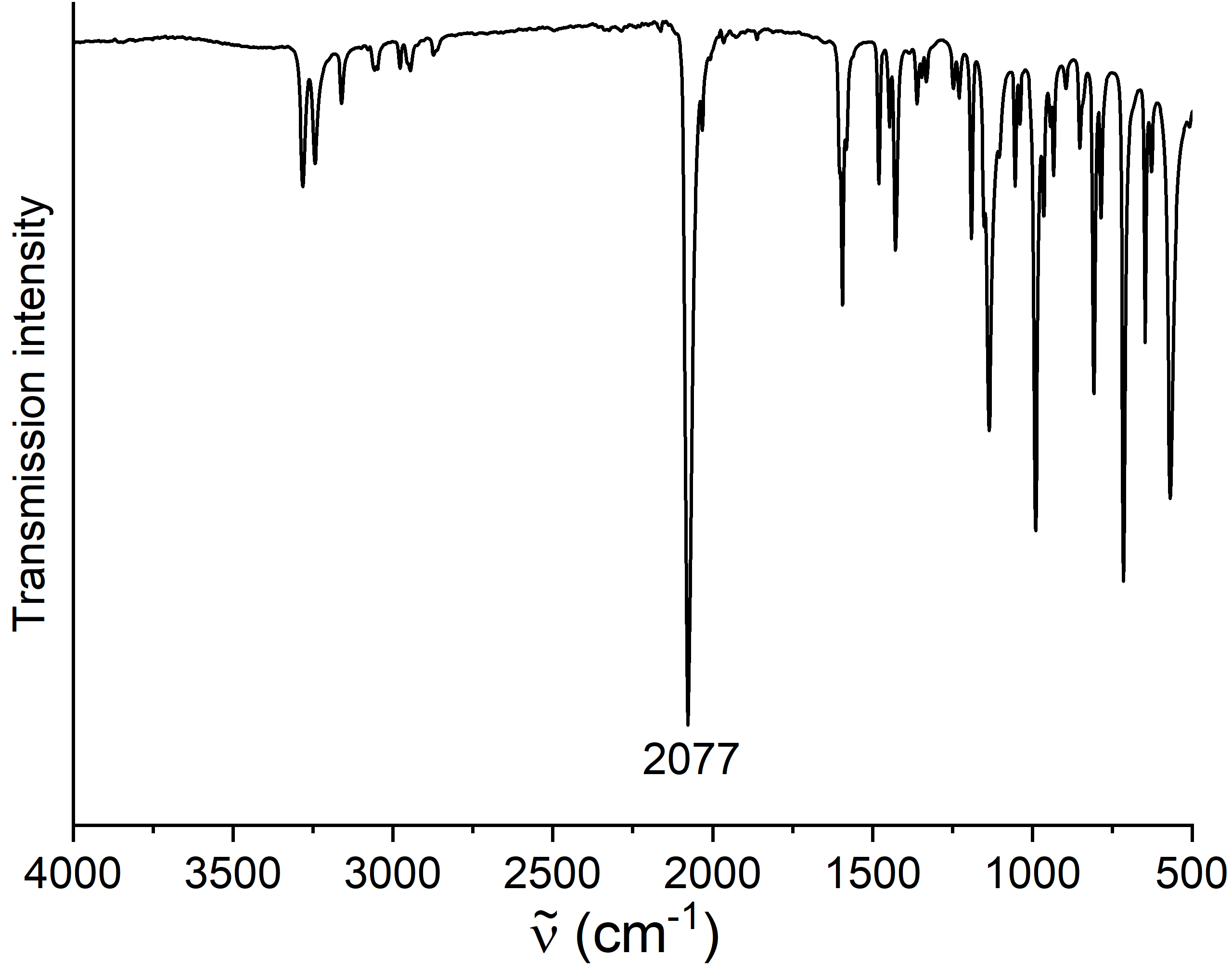

Supplement: Supplementary file 3 [file e-77-00428-sup3.png]

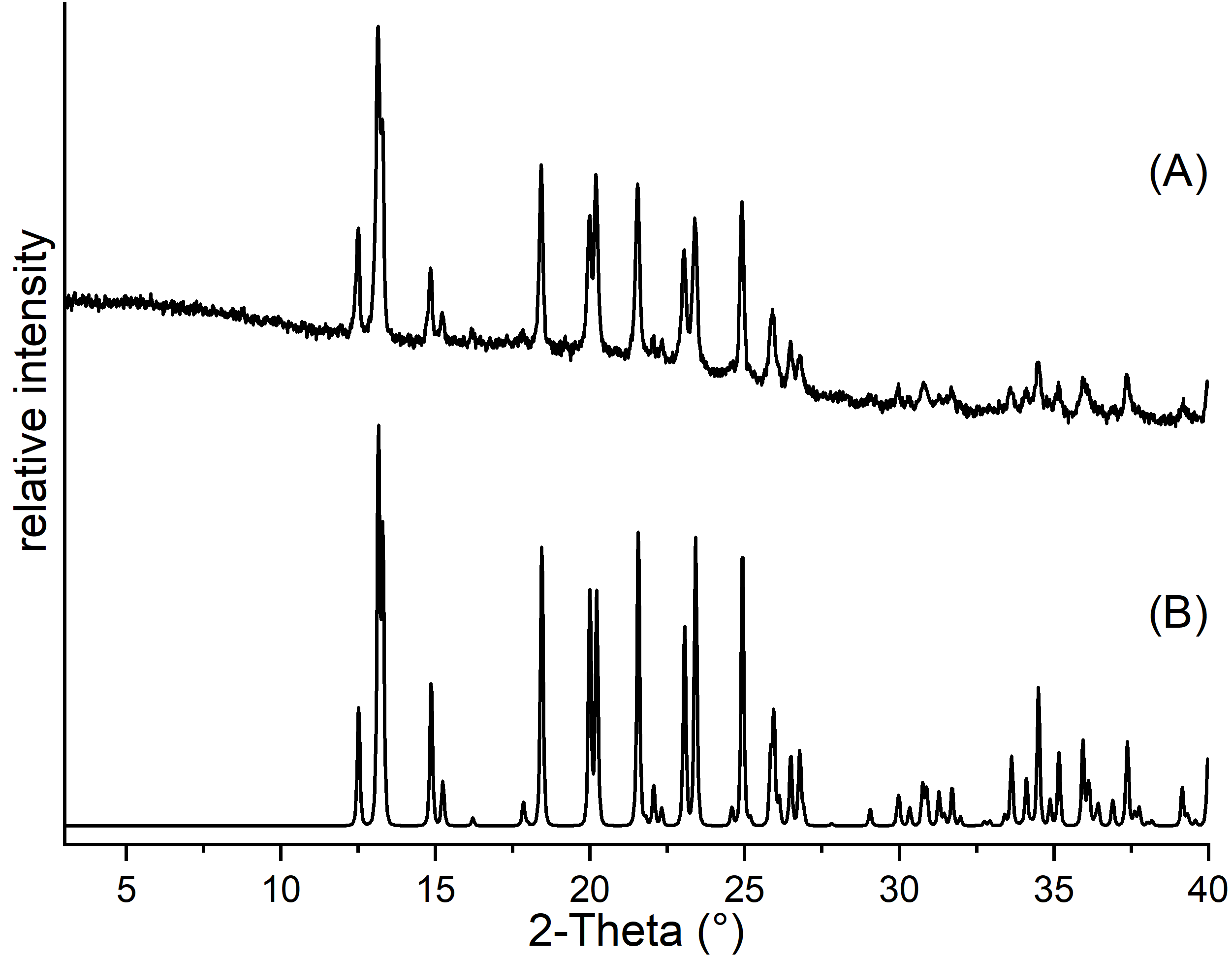

Supplement: Supplementary file 4 [file e-77-00428-sup4.png]

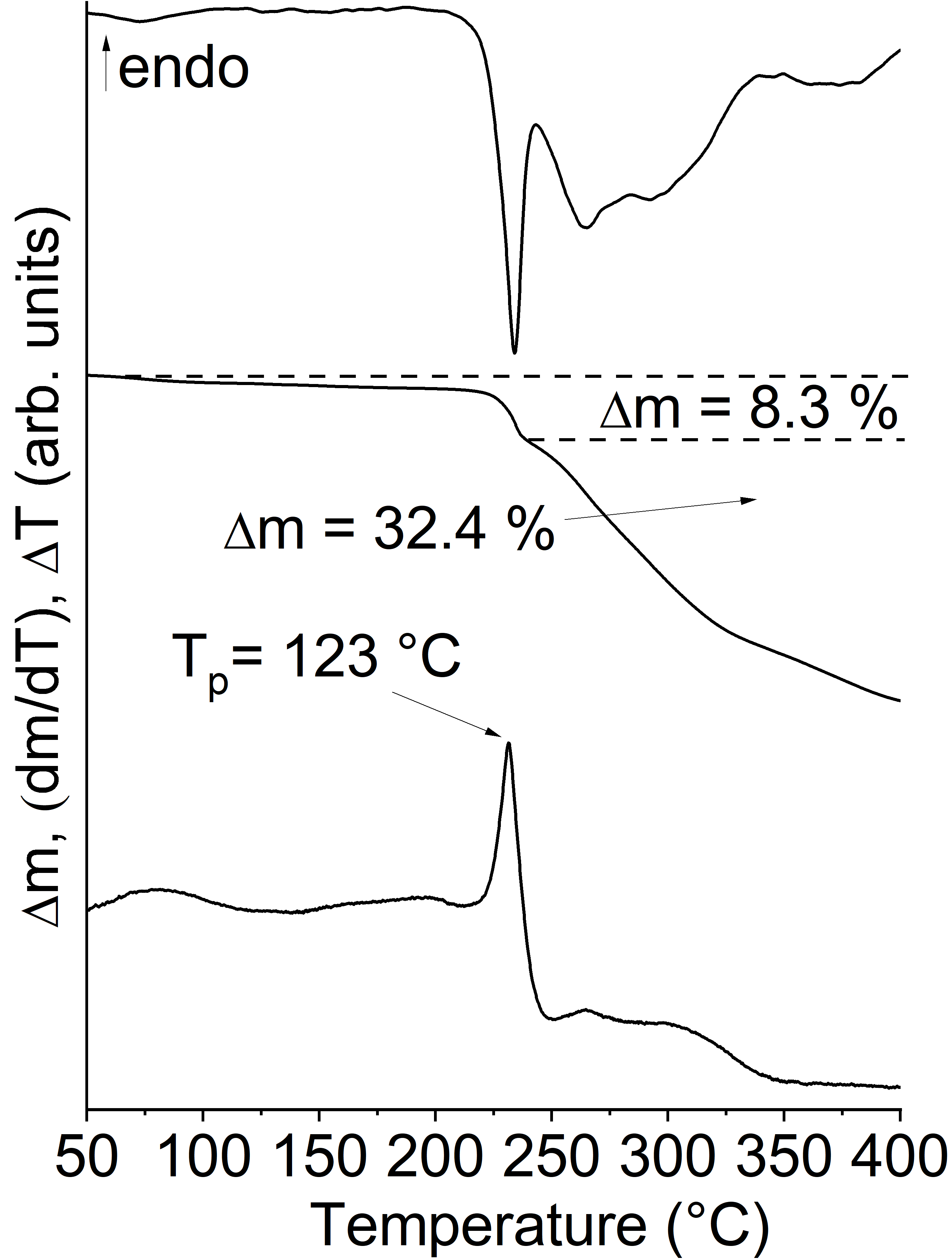

Supplement: Supplementary file 5 [file e-77-00428-sup5.png]

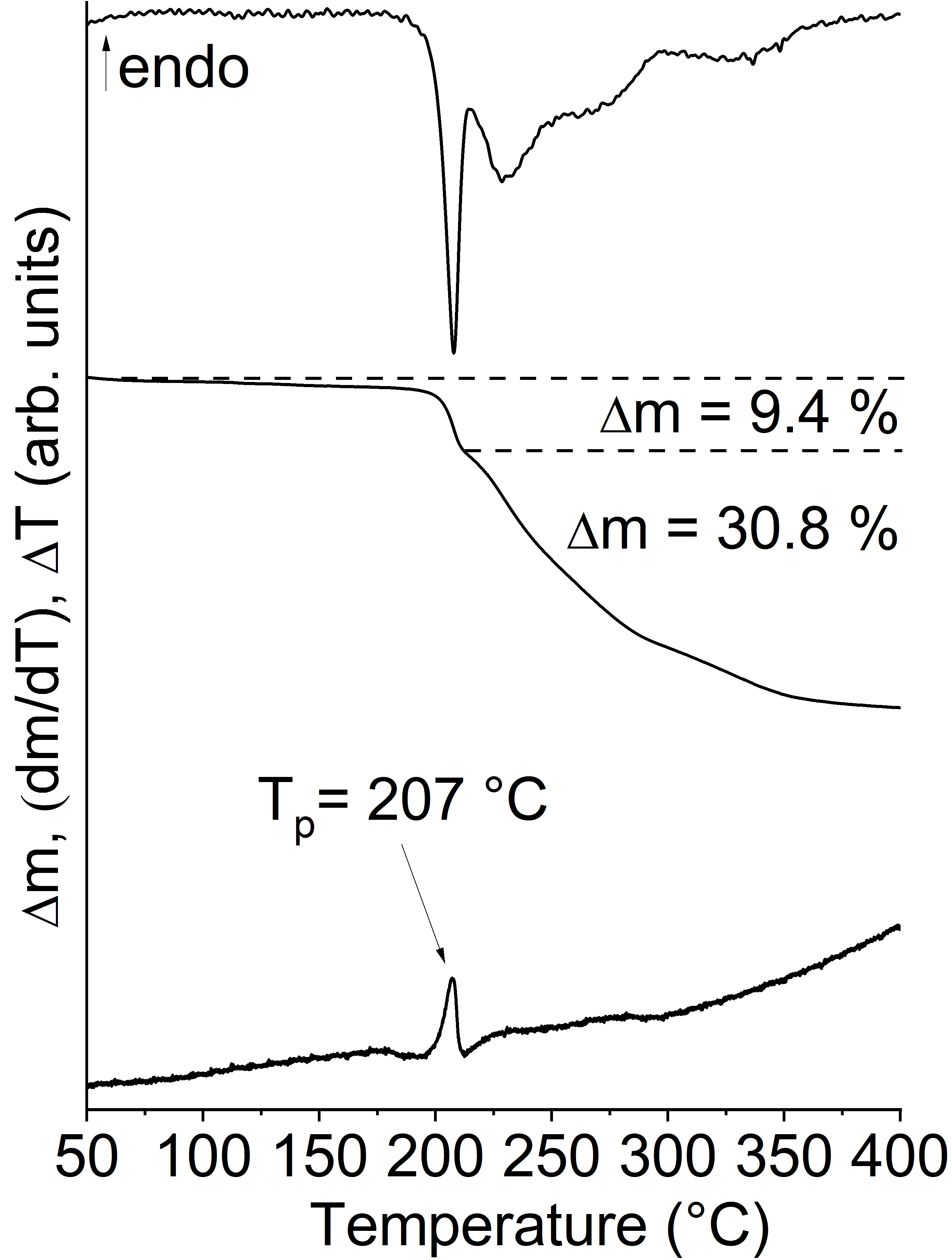

Supplement: Supplementary file 6 [file e-77-00428-sup6.png]
